# Distinctive *in-planta* acclimation responses to basal growth and acute heat stress were induced in *Arabidopsis* by cattle manure biochar

**DOI:** 10.1038/s41598-021-88856-7

**Published:** 2021-05-10

**Authors:** Abhay Kumar, Haya Friedman, Ludmila Tsechansky, Ellen R. Graber

**Affiliations:** 1 Department of Soil Chemistry, Plant Nutrition and Microbiology, Institute of Soil, Water and Environmental Sciences, Agricultural Research Organization, Volcani Center, Rishon LeZion, 7505101 Israel; 2 Department of Postharvest Science, Agricultural Research Organization, Volcani Center, Rishon LeZion, 7505101 Israel

**Keywords:** Abiotic, Heat, Environmental sciences

## Abstract

In-planta mechanisms of biochar (BC)-mediated improved growth were evaluated by examining oxidative stress, metabolic, and hormonal changes of *Arabidopsis* wild-type plants under basal or acute heat stress (–HS/ + HS) conditions with or without BC (+ BC/–BC). The oxidative stress was evaluated by using *Arabidopsis* expressing redox-sensitive green fluorescent protein in the plastids (pla-roGFP2). Fresh biomass and inflorescence height were greater in + BC(‒HS) plants than in the –BC(‒HS) plants, despite similar leaf nutrient levels, photosystem II (PSII) maximal efficiencies and similar oxidative poise. Endogenous levels of jasmonic and abscisic acids were higher in the + BC(‒HS) treatment, suggesting their role in growth improvement. HS in ‒BC plants caused reductions in inflorescence height and PSII maximum quantum yield, as well as significant oxidative stress symptoms manifested by increased lipid peroxidation, greater chloroplast redox poise (oxidized form of roGFP), increased expression of *DNAJ heat shock proteins* and *Zn-finger* genes, and reduced expression of *glutathione-S-transferase* gene in addition to higher abscisic acid and salicylic acid levels. Oxidative stress symptoms were significantly reduced by BC. Results suggest that growth improvements by BC occurring under basal and HS conditions are induced by acclimation mechanisms to ‘microstresses’ associated with basal growth and to oxidative stress of HS, respectively.

## Introduction

Adding biochar, the solid product of pyrolysis (anaerobic combustion) of carbon (C) rich biomass to the soil is often reported to have a number of agronomic benefits^1^: improved water and nutrient retention^2^, increased microbial activity and diversity^3^, and improved soil productivity and plant health^4^. Since the pyrolysis process converts C from a biosphere pool that is rapidly transformed to CO_2_ to a pool where C cycling is very slow (1000 s to tens of thousands of years), biochar is considered to have substantial C sequestration potential^5^. For these reasons, and because the pyrolysis technology can treat various organic waste streams, the pyrolysis/biochar platform has attracted considerable scientific and commercial interest.

Advances in biochar research shows that biochar application as a soil amendment can significantly change soil physicochemical and biological properties^6^. Some of the changes can lead to increasing crop growth and yield^7^, for example by biochar-induced increases of water holding capacity^2^, topsoil nutrient availability and retention^8^ and increased topsoil organic matter contents^9^. Biochar can also immobilize or remediate various soil contaminants^10^, control pesticide toxicity^11^, regulate pH and nutrient availability in acidic/alkaline/saline and nutrient deficient soils^1^, and assist in improving soil fertility^12^. Biochar application to soil may also increase fertilizer use efficiency in crops^13^. Some of these impacts have been attributed to changes in rhizosphere microorganism communities^14^, an increase in the free energy of specific nutrient transport into root cell membranes due to redox active functionalized groups^15^, and release of water-soluble C nanoparticles that enhance water and nutrients uptake capacity^16^. Various biochar-related mechanisms may play a role, as recently reviewed in Joseph, et al.^17^.

One intriguing feature of biochar addition to soil was the discovery that it can potentiate system-wide plant defense responses (induced resistance) against diseases caused by plant pathogens^18,19^. Analysis of defense-related gene expression showed that biochar can activate both salicylic acid (SA)-mediated systemic acquired resistance (SAR) and jasmonic acid (JA)/ethylene (ET)-mediated induced systemic resistance (ISR) pathways^20,21^ against pathogen attack. Like biotic stresses, abiotic stresses induce system-wide plant responses along varied metabolic pathways, some of which are similar to those invoked against biotic stresses^22^. Abiotic stresses result from environmental pressures: temperature extremes (heat or cold), water shortages or excesses, soil toxins (salts, metals or organic pollutants), and others^23^. In the face of environmental pressures, plants rapidly regulate their cellular mechanisms to maintain homeostasis; this process is known as acclimation.

Only a small number of studies have examined *in-planta* responses to biochar when faced with environmental pressures^6,24,25^ that are not soil toxins. Under both sufficient and drought water conditions, *Chenopodium quinoa* grew significantly better in biochar treatments^26^. This was suggested to be due to improved plant traits (lower proline content and less negative osmotic potential) rather than to increased root zone water content^26^, an effect mirrored in pot trials with maize^27^. Improved pepper plant productivity in biochar treatments throughout a multi-year field trial conducted under extreme environmental pressures (high evaporation demand and vapor pressure deficit, high daytime temperatures (heat stress) at planting and low nighttime temperatures at fruiting, brackish water irrigation) raised the question of whether the improved productivity was an outcome of biochar-elicited acclimation responses^4^ in the plants. The present study was designed to test this question directly using heat stress (HS) in *Arabidopsis* growing in a medium with or without biochar under well-controlled basal conditions. *In-planta* responses were specifically examined because there is very little information about them. If BC does affect plant responses to HS, this could be an impactful result because HS is one of the most serious abiotic environmental threats to plants. HS is becoming more common due to climate change in many parts of the world.

HS is a potentially deadly event for a plant. An immediate outcome of HS includes increased generation of reactive oxygen species (ROS)^28^. ROS cause acute damage to the cellular machinery by reaction with membrane lipids and proteins and alteration of metabolic activity^29,30^. Chloroplasts are one of the major sites of ROS production and are intimately associated with the development of oxidative burst^28,31^.

Plants rapidly activate counteractive defense measures following heat-induced ROS production because of the deadly destruction ROS can wreak on plant tissues. The complex signaling network regulating the response of plant cells to HS is activated in large part by HS transcription factors A (HsfA)^32^. These regulators stimulate the expression of various genes that produce proteins directly and indirectly involved in defense and stress adaptation^33^. Among them, proteins such as heat shock proteins (Hsp)^34^, Zn-finger (ZnF)^35^, and glutathione-*S*-transferase (GST)^36^ are known for their key functions in various abiotic stress responses to HS. Hsp act as molecular chaperones; their presence in the cellular vicinity prevents protein denaturation and alleviates cytotoxic effects^37^. ZnF protein has roles in transcription and protein synthesis mechanisms^38^, while GST regulates the cellular detoxification of a variety of electrophilic moieties and toxic substances generated by ROS^39^. Metabolic events activated by HsfA signaling pathways include regulation of phytohormones like JA, SA^40^, abscisic acid (ABA), auxins^41^, and cytokinins^42^. Phytohormone levels regulate subsequent plant responses to HS in order to avoid its deleterious effects on the plant's basic physiology^43,44^. Studies indicate that changes in plant hormonal balances trigger several signaling cascades related to cellular defense mechanisms^45^.

**Figure 1:**
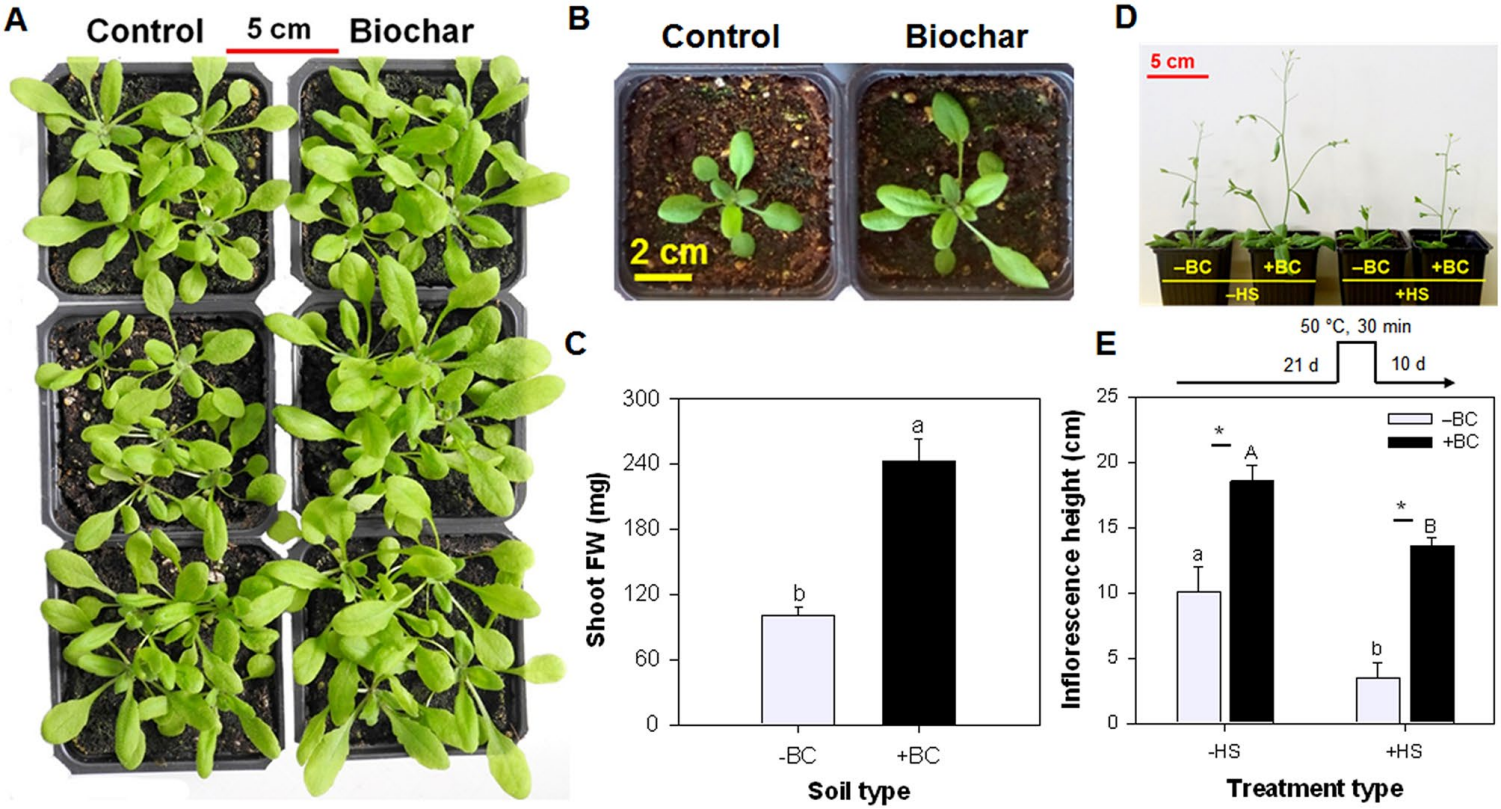
Effect of absence (‒BC or Control) and presence (+ BC or Biochar) of biochar on *Arabidopsis thaliana* seedlings growth. (**A**) Plant growth; (**B**) Rosette growth; (**C**) Shoot fresh weight after 21 d of seed germination; (D) Inflorescence height in plants in ‒HS and + HS conditions at 21 days and grown for additional 10 days. Images of plant growth and rosette growth were captured after 21 d and inflorescence height was captured after 31 d of seed germination. Data are representative of multiple repeated experiments. Columns (n = 3; means ± S.E.) labeled by a different letter in Figure C are significantly different at *p* ≤ 0.05. Columns (n = 3; means ± S.E.) labeled by different lowercase and uppercase letters in Figure E are significantly (*p* < 0.05) different within the ‒BC and + BC groups and in ‒HS and + HS treatments, respectively. Asterisk denotes significant difference at *p* ≤ 0.05 according to Tukey Kramer HSD test between –BC and + BC at a given heat treatment.

Examining plant acclimation responses to acute HS and the impact of biochar (BC) in the growing medium on those responses was the goal of the present work, using *Arabidopsis thaliana* as a model plant due to the availability of genetic material, genomic information, and mutants expressing redox-sensitive green fluorescent protein in the plastids. The study examined inflorescence emergence, lipid peroxidation, phytohormones, and the expression of heat shock responsive genes. In addition, plants expressing redox-sensitive green fluorescence protein (roGFP) in plastids^46^ were employed to examine plastid-specific redox poise modification. Four treatments were tested: –BC(–HS), + BC(–HS), –BC(+ HS), and + BC(+ HS), whereby BC was pre-mixed in the growing medium at 10 or 0 g Kg^‒1^ by dry weight (+ BC and –BC, respectively) at the beginning of the experiment. Acute heat stress or mock stress (+ HS and –HS, respectively) was applied 21 days following seed germination. The BC was produced at a highest treatment temperature (HTT) of 450 °C from cattle manure feedstock. A previous study reported the results of a detailed chemical and spectroscopic investigation of this biochar^47^. Scanning electron microscopy and energy dispersive X-ray spectroscopy analysis revealed it has a complicated surface structure consisting of a porous C-matrix with minerals and inorganic compounds in the pores (see Fig. 1A and B of Kumar, et al.^47^). This accords with the high proportion of cellulosic plant residues in the cow manure. The biochar had a high concentration of aromatic C, C‒O, and C‒N = C groups in X-ray photoelectron spectroscopy (XPS) analysis, but a low total organic C content of 22.7%^47^.

## Results

### Plant growth and physiological parameters

Overall plant growth in the + BC(‒HS) treatment was significantly better than in the –BC(‒HS) treatment, as seen in the size of the rosettes 21 days after germination (Fig. 1A, B) and shoot fresh weight (Fig. 1C). Biochar furthermore had a significant impact on inflorescence height at 31 days post-germination, whereby + BC(‒HS) inflorescence was 1.8-fold higher than –BC(‒HS) inflorescence height (Fig. 1D, E). There was no significant impact of biochar on leaf mineral nutrient content (Table 1).

**Table 1:** Effect of absence (‒BC) and presence (+ BC) of biochar on the nutrient element levels of *Arabidopsis thaliana* plants grown for 21 days.

Nutrient elements	Treatment	
	‒BC	+ BC
Zinc (mg Kg^–1^)	71 ± 5.9^a^	75 ± 1.6^a^
Iron (mg Kg^–1^)	207 ± 31^a^	198 ± 37^a^
Copper (mg Kg^–1^)	ND	ND
Manganese (mg Kg^–1^)	47 ± 2.1^a^	38 ± 1.8^a^
Magnesium (g Kg^–1^)	6.4 ± 0.1^a^	6.1 ± 0.2^a^
Calcium (g Kg^–1^)	126 ± 3.5^a^	121 ± 2.4^a^
Potassium (g Kg^–1^)	66 ± 1.5^a^	70 ± 1.4^a^
Sodium (mg Kg^–1^)	37 ± 2.8^a^	40 ± 2.8^a^
Nitrogen (g Kg^–1^)	4.1 ± 0.4^a^	3.8 ± 0.1^a^

Data presented are mean ± standard error (*n* = 3) and different letters indicate significant (*p* ≤ 0.05) differences with each other (‒BC and + BC) (ANOVA, Tukey test). ND—not detected.

HS applied at 21 days was found to significantly reduce (by 65%) inflorescence determined at 31 days in the –BC(+ HS) treatment compared with the –BC(–HS) condition (Fig. 1D, E). The interaction between + BC and + HS demonstrated that the presence of biochar helped to protect inflorescence against the deleterious impact of the heat (Fig. 1D, E). There was a significant increase in inflorescence height (3.9-fold) in + BC(+ HS) plants in comparison to –BC(+ HS) plants (Fig. 1E). The presence of biochar did not completely eliminate the impact of HS on inflorescence growth: 18.5 ± 1.2 cm in + BC(‒HS) versus 13.6 ± 0.6 cm in + BC(+ HS) treatments.

**Figure 2:**
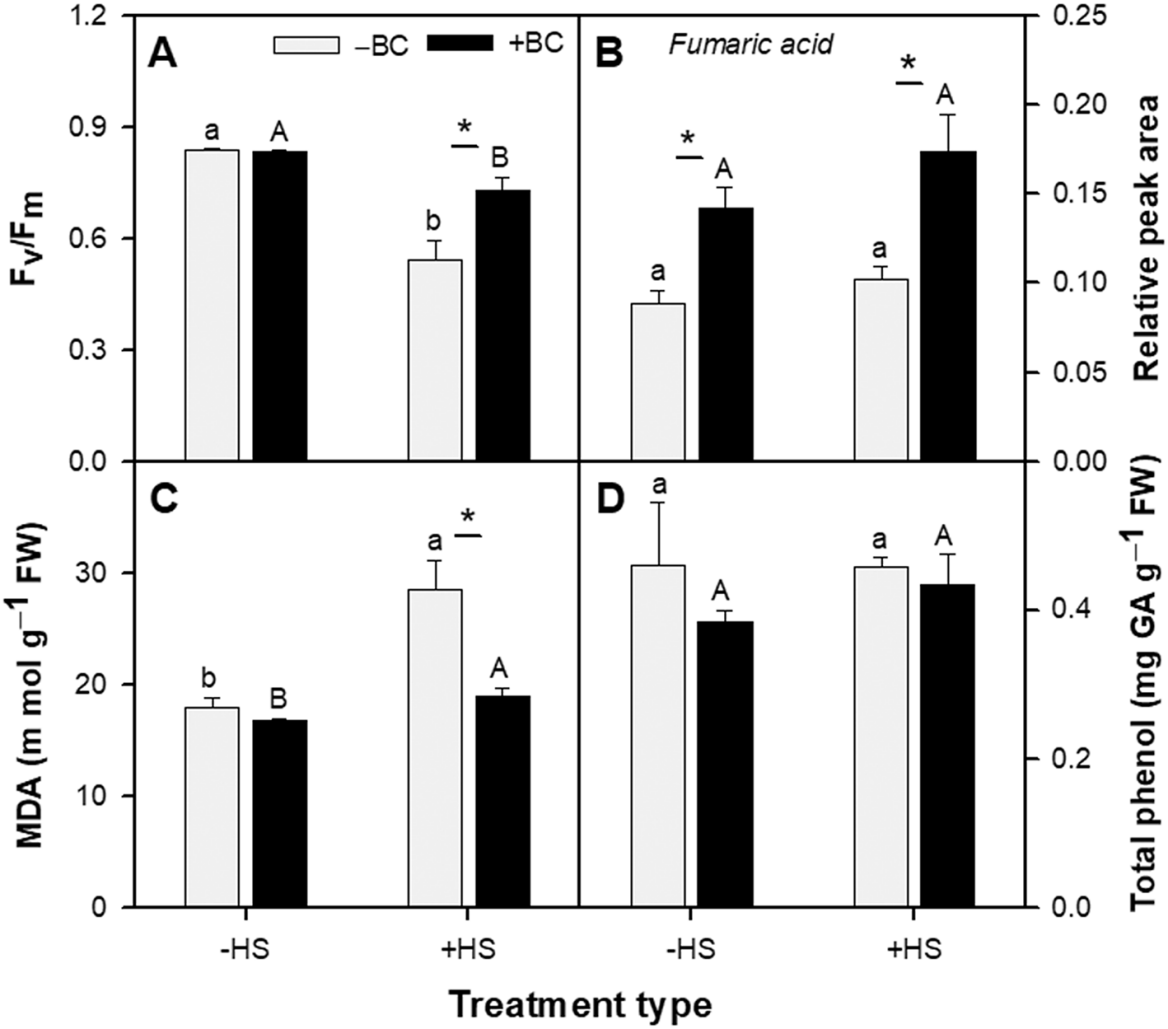
Effect of absence (‒BC) and presence (+ BC) of biochar on physiological parameters of *Arabidopsis thaliana* in ‒HS and + HS conditions at 21 days. (**A**) Quantum yield of photosystem II (F_v_/F_m_); (B) Fumaric acid contents; (C) Malondialdehyde (MDA) concentration as representative of lipid peroxidation; and (D) Total phenolic level. Data in A and C panes are representative of multiple repeated experiments, and in B and D of one repeated experiment. Columns (n = 3; means ± S.E.) labeled by different lowercase and uppercase letters are significantly (*p* < 0.05) different within the ‒BC and + BC groups and in ‒HS and + HS treatments, respectively. Asterisk denotes significant difference at *p* ≤ 0.05 according to Tukey Kramer HSD test between –BC and + BC at a given heat treatment.

There was no significant difference in PSII maximum quantum yield (F_v_/F_m_) in –BC(‒HS) and + BC(‒HS) treatments, while F_v_/F_m_ levels in –BC(+ HS) plants were significantly lower than in –BC(‒HS) plants (Fig. 2A; Table S3). F_v_/F_m_ levels were significantly higher in + BC(+ HS) versus –BC(+ HS) plants although the presence of BC did not completely restore the stressed plant photochemical efficiency to that of the healthy unstressed plant: 0.84 ± 0.005 in + BC(‒HS) versus 0.73 ± 0.03 in + BC(+ HS) treatments.

Concentrations of fumaric acid (FA) in the *Arabidopsis* shoots were significantly higher (by 1.7-fold) in + BC compared with –BC treatments, irrespective of HS (Fig. 2B). FA is indicative of mitochondrial function. There was no significant difference in lipid peroxidation (as estimated by malondialdehyde (MDA) concentrations) in shoots of the –BC(‒HS) and + BC(‒HS) treatments (Fig. 2C). HS resulted in a significant increase in lipid peroxidation, showing a 1.6 fold increase in MDA in ‒BC(+ HS) compared with –BC(‒HS). The presence of biochar significantly protected the plants against lipid peroxidation caused by HS, showing much reduced MDA concentrations (19 ± 0.6 compared with 28.5 ± 2.6 units in + BC(+ HS) versus –BC(+ HS), respectively) (Fig. 2C; Table S3). Levels of total phenols which includes polyphenol antioxidants^48^ in all treatments were similar (Fig. 2D). There was some evidence for the influence of the various treatments on shoot fatty acids (Supplemental Figure S1). + BC(–HS) caused an increase in the relative abundance of octadecanoic acid in the –BC(–HS) treatments (Supplemental Figure S1D). Octadecanoic acid was also increased due to HS in the –BC treatments (Supplemental Figure S1D; –BC(–HS) versus –BC(+ HS)). There was a decrease in the relative abundance of the more highly unsaturated C16 fatty acids, 7, 10-hexadecadienoic acid (Supplemental Figure S1B) and 7,10,13-hexadecatrienoate (Figure S1C), in the + BC(+ HS) treatment compared with the –BC(+ HS) treatment. The mono-unsaturated 9-hexadecenoic acid was unchanged by any treatments (Supplemental Figure S1A). The + BC(+ HS) treatment resulted in an increase in the monounsaturated 9-octadecenoic acid (Supplemental Figure S1E) and a decrease in the tri-unsaturated C18 acid, 9,12,15-octadecatrienoic acid (Supplemental Figure S1G) compared with the + BC(–HS) treatment. Neither biochar nor HS cased any signifcant changes in the di-unsaturated 9,12-Octadecadienoic acid (Supplemental Figure S1F).

### Plastid redox status as determined in-vivo on whole plants harboring the oxidation sensitive roGFP

**Figure 3:**
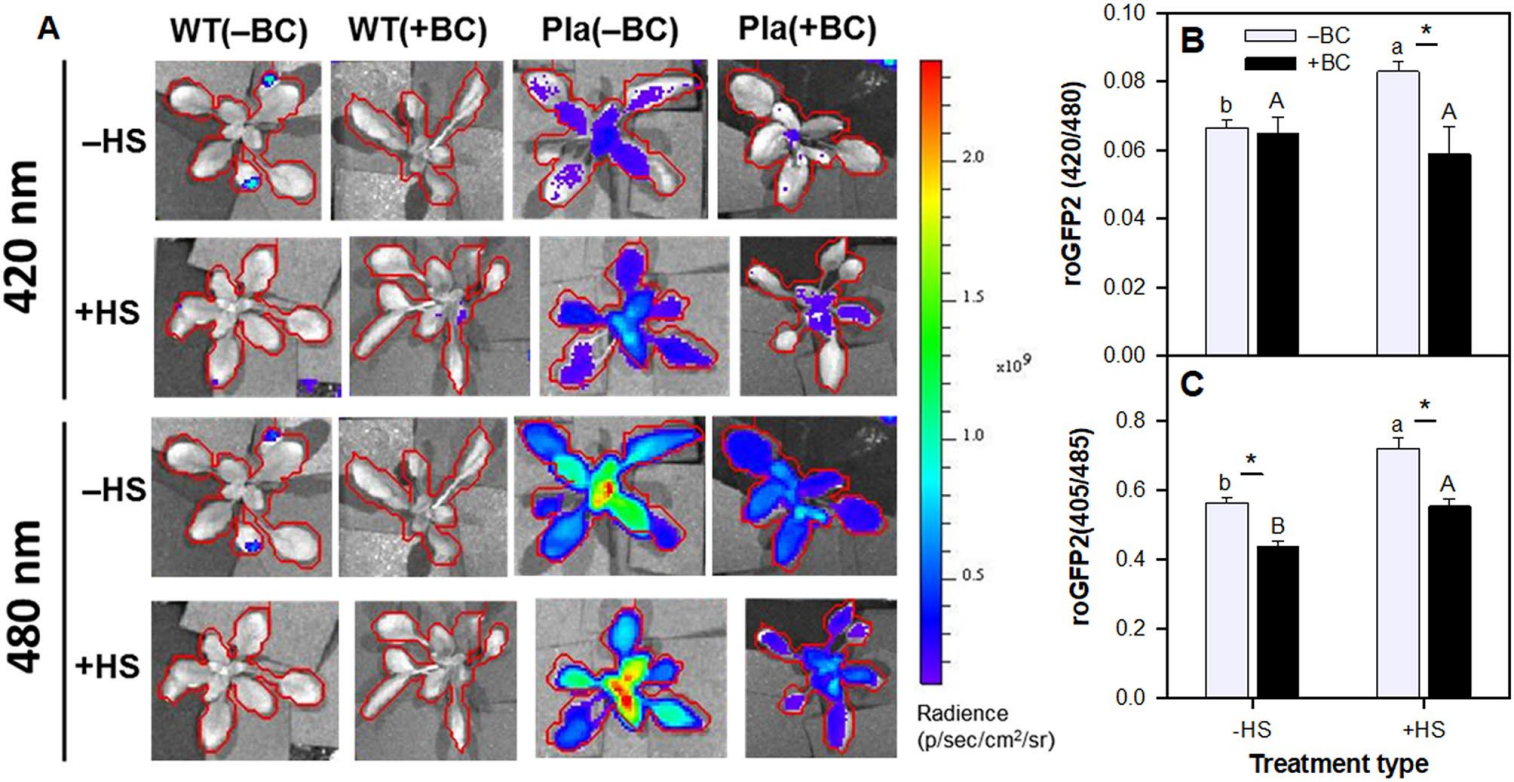
Effect of absence (‒BC) and presence (+ BC) of biochar on the redox level of wild type (WT) plants and *Arabidopsis thaliana* expressing roGFP2 in the plastids (pla-roGFP2) under ‒HS and + HS conditions at 21 days. (**A**) *In-vivo* qualitative depiction of whole WT and roGFP2 plants excited at 420 nm (oxidation) and 480 nm (reduction); (**B**) *In-vivo* redox (oxidized/reduced) state of roGFP2 plants calculated after subtracting WT values from respective roGFP2 plants; (**C**) *In-vitro* redox (oxidized at 405 nm /reduced at 485 nm) state of roGFP2 plants analyzed using leaf discs and calculated after subtracting WT values from respective roGFP2 plants. Data represents three combined separate experiments. Columns (n = 9; means ± S.E.) labeled by different lowercase and uppercase letters are significantly (*p* < 0.05) different within the ‒BC and + BC groups and in ‒HS and + HS treatments, respectively. Asterisk denotes significant difference at *p* ≤ 0.05 according to Tukey Kramer HSD test between –BC and + BC at a given heat treatment.

The oxidative status of roGFP indicates the plastid redox poise and is expressed in the 420/480 fluorescence ratio. A higher ratio indicates higher oxidative state. The ratios of 420/480 in –BC(‒HS), + BC(‒HS), and + BC(+ HS) treatments were very similar, while the 420/480 fluorescence ratio in the –BC(+ HS) treatment was significantly greater (Fig. 3A, B). Hence, –BC(+ HS) treatment increased plant oxidation, while the + BC(+ HS) treatment exhibited plant oxidation at the same level as the basal condition. The *in-vitro* study using small leaf discs gave results similar to those of the whole plant *in-vivo* study, with the exception of the –HS condition (Fig. 3C); the dissimilarity likely reflects the difference between examining the whole live plant versus small leaf discs.

### Relative expression of stress-responsive genes

**Figure 4:**
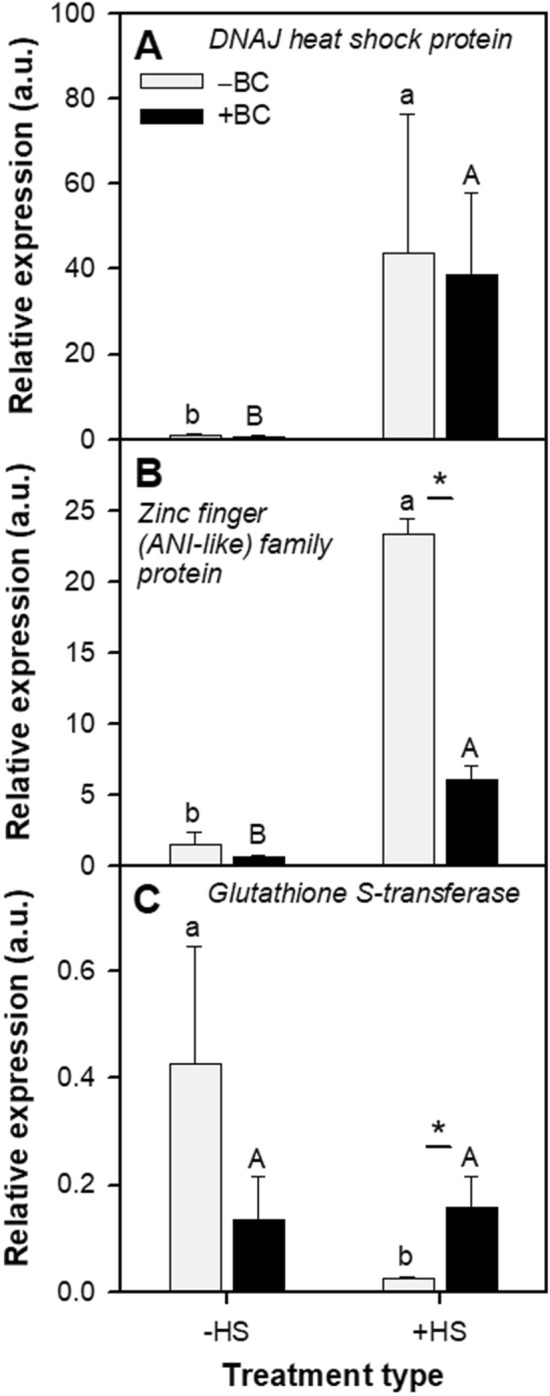
Effect of absence (‒BC) and presence (+ BC) of biochar on the relative expression of analyzed genes of *Arabidopsis thaliana* in ‒HS and + HS conditions at 21 days. (**A**) *DNAJ heat shock protein* (*Hsp40*); (**B**) *Zinc finger* (*ANI-like*) *family protein* (*ZnF*) and (**C**) *glutathione S-transferase* (*GST*). Data are representative of two repeated experiments. Columns represents the mean value (n = 3) and range (calculated by evaluating using ΔΔCT ± S.E.)^103^.

Of the five genes examined, the relative expression of three of them, *heat shock protein 40* (*Hsp40*) or (*DNAJ*), *zinc family protein* (*ZnF)*, and *glutathione-S-transferase* (*GST*), demonstrated some significant changes in response to the treatments (Fig. 4). + BC(–HS) treatment had no effect on those genes when compared with–BC(–HS) treatment (Fig. 4). However, the ‒BC(+ HS) condition had significantly upregulated relative expression of *Hsp40* by 39-fold (Fig. 4A) and of *ZnF* by 16-fold (Fig. 4B), and downregulated relative expression of *GST* gene by 17-fold (Fig. 4C) as compared with the –BC(–HS) condition. The relative expression of *Hsp40* was no different under + BC(+ HS) and –BC(+ HS) conditions (Fig. 4A). On the other hand, the relative expression of *ZnF* was significantly lower (Fig. 4B) and *GST* was significantly higher in + BC(+ HS) compared with –BC(+ HS) plants (Fig. 4C), showing that BC had an impact on plant stress-related gene responses under HS. Neither biochar nor HS had any impact on the relative expression of other stress-related genes: *cytochrome P450-like protein* (*CP450)* and *glycosyltransferase* (*GTF*) (Supplemental Figure S2).

### Plant hormone responses

**Figure 5:**
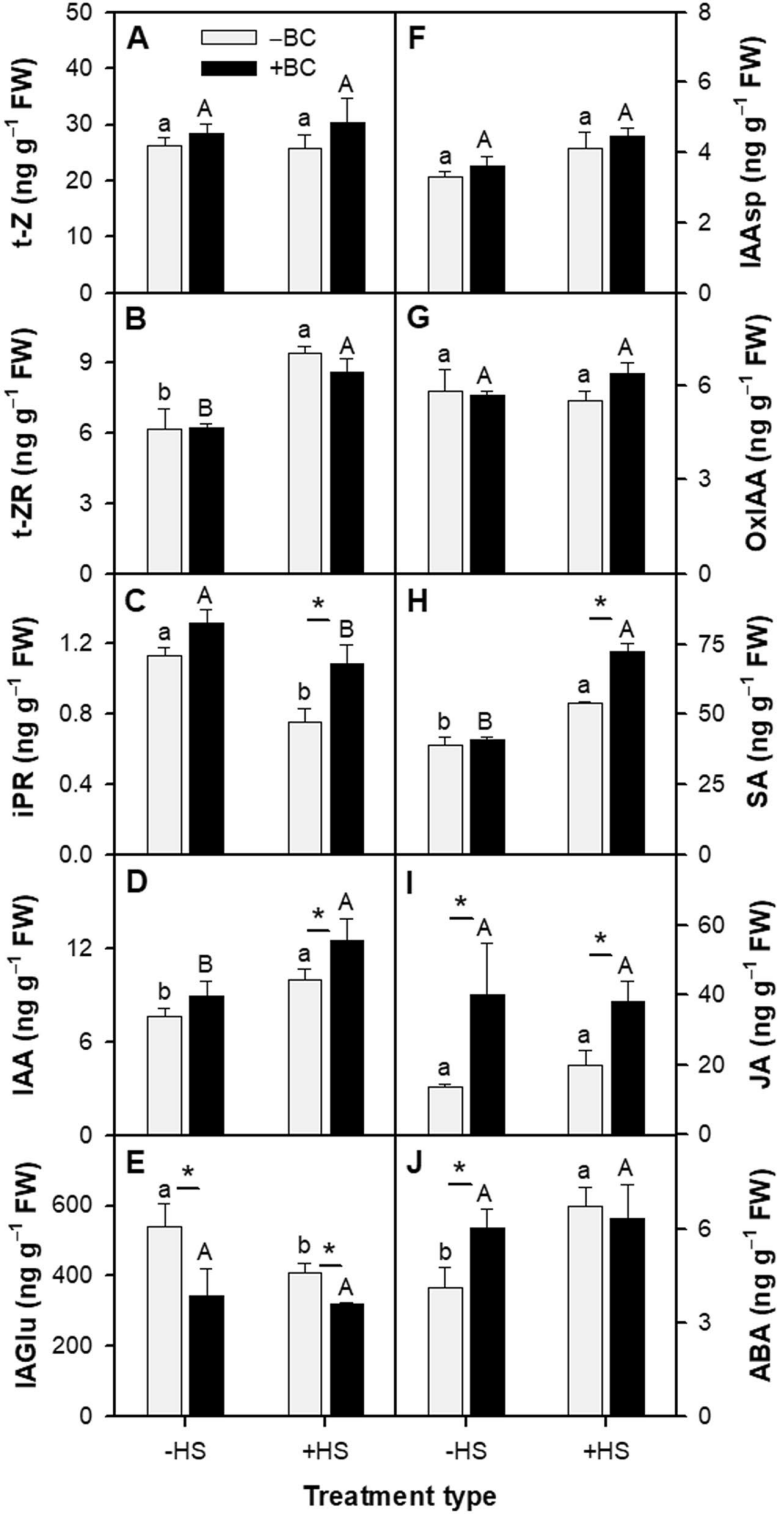
Effect of absence (‒BC) and presence (+ BC) of biochar on the plant hormones level of *Arabidopsis thaliana* in ‒HS and + HS conditions at 21 days. (**A**) *trans*-Zeatin (t-Z); (**B**) Isopentenyladenine (iP); (**C**) Isopentenyladenosine (iPR); (**D**) indole-3-acetic acid (IAA); (**E**) Indole-3-acetyl glutamic acid (IAAGlu); (**F**) Indole-3-acetylaspartic acid (IAAsp); (**G**) Oxindole-3-acetic acid (OxIAA); (**H**) Salicylic acid (SA); (**I**) Jasmonic acid (JA); and (**J**) Abscisic acid (ABA). Data represents two combined separate experiments. Columns (n = 6; means ± S.E.) labeled by different lowercase and uppercase letters are significantly (*p* < 0.05) different within the ‒BC and + BC groups and in ‒HS and + HS treatments, respectively. Asterisk denotes significant difference at *p* ≤ 0.05 according to Tukey Kramer HSD test between –BC and + BC at a given heat treatment.

Hormones contribute to plant growth and ROS tolerance^45,49^. BC and HS variously modified the levels of auxins, cytokinins, ABA, JA and SA and some of their derivatives (Fig. 5; Table S3). Non-biochar treated plants responded to HS (–BC(+ HS)) with significantly increased levels of trans-zeatin riboside (t-ZR; Fig. 5B), indole-3-acetic acid (IAA; Fig. 5D), SA (Fig. 5H) and ABA (Fig. 5J), and decreased levels of isopentenyladenosine riboside (iPR; Fig. 5C) and indole-3-acetyl-l-glutamic acid (IAGlu; Fig. 5E) when compared to the –BC(–HS) treatment. Under non-stressed basal conditions, the plants growing in biochar had elevated levels of JA (Fig. 5I) and ABA (*p* ≤ 0.055) (Fig. 5J), and lower levels of IAGlu (Fig. 5E) (+ BC(‒HS) in comparison to –BC(‒HS) treatments). The decline in iPR levels due to HS in –BC plants was less in the + BC plants (Fig. 5C), and the increases in IAA (Fig. 5D) and SA (Fig. 5H) levels due to HS in –BC plants were yet greater in + BC(+ HS) plants. There were no significant changes in the plants' trans-zeatin (t-Z; Fig. 5A), indole-3-acetylaspartic acid (IAAsp; Fig. 5F), and oxindole-3-acetic acid (OxIAA; Fig. 5G) levels due to any treatment (BC or HS).

## Discussion

Biochar addition had no impact on plant nutrient levels or photochemical functions. Yet, it did improve biomass and inflorescence growth via inducing key biochemical and hormonal levels under basal (‒HS) growth conditions, indicating that biochar had a role in modifying the physiological status of the plant. The impact of the biochar on plant physiology was further exemplified by its effect on plant responses to acute HS. Symptoms of HS in *Arabidopsis* without BC (‒BC) included decreased inflorescence growth and increased oxidative status; these HS-induced responses were reduced in plants grown in the + BC treatment. Altogether, the results are suggestive that the presence of BC induced a type of acclimation of the plant to every day “microstresses” that occur during growth under basal conditions. This state can be thought of as a 'biochar-elicited early acclimated state’ against everyday microstresses. This early-acclimated state primed the plants to cope better with the subsequent acute heat stress: a ‘biochar-elicited acclimated state’.

**Figure 6:**
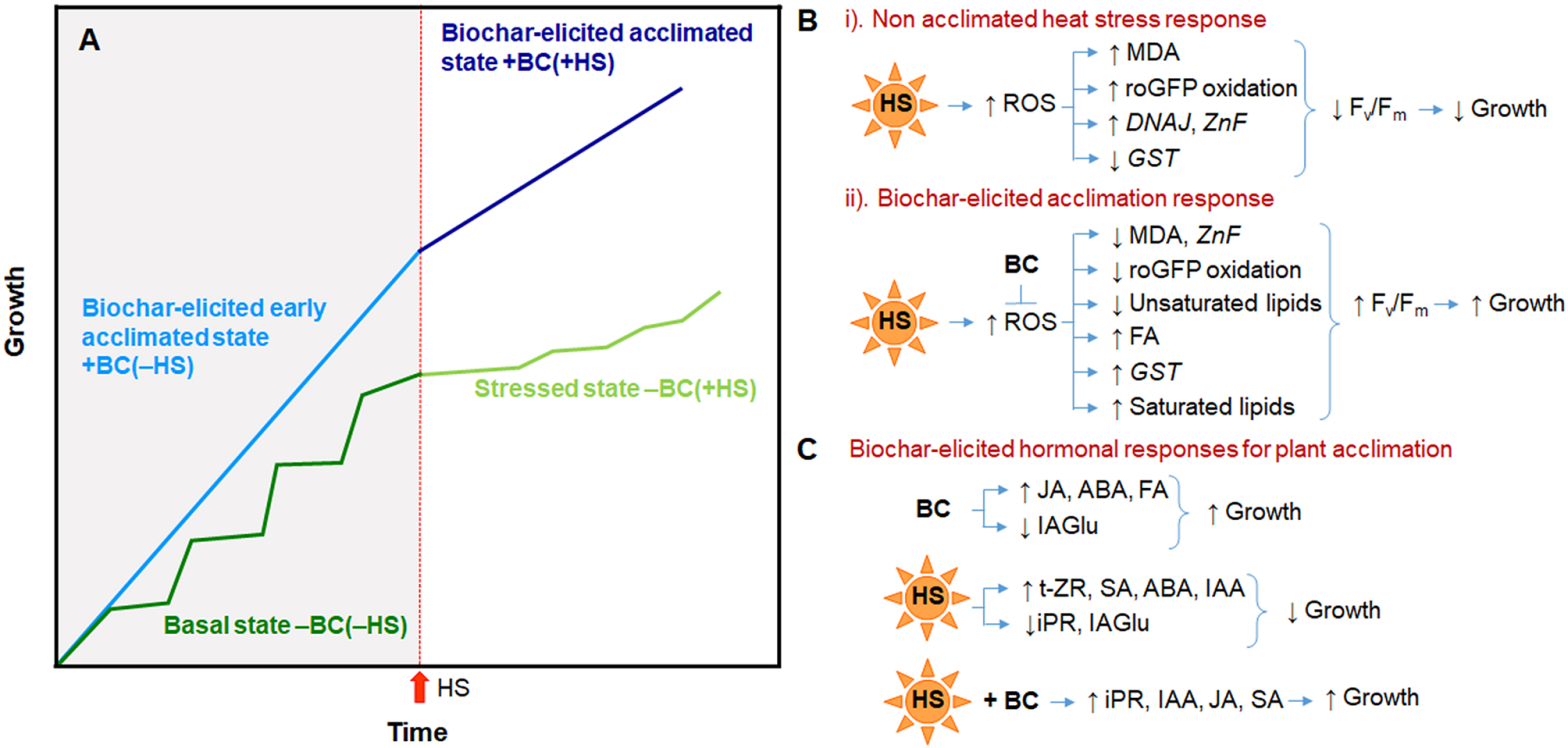
Schematic depictions (**A**) of the impact of biochar on plant performance, and (**B, C**) summary of in-planta responses. (**A**) The dark green line, corresponding to the ‒BC(‒HS) treatment, represents basal plant growth under the given environmental conditions. The step-wise pattern represents everyday microstresses that a plant experiences throughout its normal growth or life cycle. An acute heat stress, labeled HS, occurs at some time. Following the HS, the plant’s basal growth and development is impaired (light green line). This corresponds to the ‒BC(+ HS) treatment. The solid light blue line represents the “biochar-elicited early acclimated state”, wherein plant growth and development is enhanced compared with the basal state, and represents the + BC(‒HS) condition. This state involves a boosted TCA cycle and elevated JA and ABA hormone levels. The dark blue line represents a “biochar-elicited acclimated state”, corresponding to the + BC(+ HS) condition. This state involves ROS-related oxidative stress responses. (**B, C**) In-planta responses in the biochar-elicited early-acclimated state, heat stressed state, and biochar-elicited acclimated state compared with the basal state.

By ‘biochar-elicited early acclimated state’, we refer to improved plant performance in the face of everyday, non-acute stresses, as compared with a conventional acclimated state that may ensue following a major acute stress such as the HS applied in this study (Fig. 6). The relationship between the ‘BC-elicited early acclimated state’ and the ‘basal state’ (no biochar) of the plant is depicted in the schematic model in Fig. 6. In the absence of an acute stress, the ordinary growth rate of plants in the basal state will exhibit a type of stair-step pattern. This pattern, originally proposed by Thompson, et al.^50^, represents small, everyday stresses, or “microstresses”, that will result in short-lived slowdowns in growth rate that are part of an overall increase in growth over time. In the BC-elicited early acclimated state, the effect of microstresses is lower and thus the overall rate of growth is higher than in the basal state. We suggest that a plant in the ‘biochar-elicited early acclimated state’ is ‘primed’ to activate rapid responses to an acute stress, in this case, heat shock. Thus, plant growth of BC -treated plants continues to be much better following the HS than growth of non-BC treated plants. The biochar-elicited early-acclimated state is somewhat different from a traditional ‘primed state’, wherein plants can be 'primed' by various stimuli, such as chemical, biological, or environmental agents^51^, to respond with fast and strong defenses if faced by a future acute biotic or abiotic stress^52^. Usually, priming reduces the growth of the plant^53^, but our study shows the opposite: growth in the biochar-elicited early-acclimated state is better than in the basal state. This effect appears to parallel reports of superior plant performance and rapid systemic resistance responses to acute biotic stresses^4,21,54,55^ in plants grown in potting media supplemented by biochar.

It seems that enhanced TCA cycle activity, as exemplified by the increase in fumaric acid levels and elevated JA and ABA levels, may be involved in the alleviation of the plant microstresses and contribute to an early acclimated state of the biochar-amended plants. Elevated plant JA and ABA levels are frequently taken as evidence of stress^56–58^, however, stress would not be expected to correspond to the improved growth and flowering observed in + BC(‒HS) plants as compared with –BC(‒HS) plants^59,60^. Likewise, if biochar were to induce stress, reduced photosynthetic efficiency, increased lipid peroxidation or increase in oxidative status could be anticipated^61^, yet, this is not the case in + BC(‒HS) plants compared with –BC(‒HS) plants. It has been seen previously that exogenous application of JA and to a lesser degree also ABA elicited improved tolerance to various stresses in multiple plants^62,63^. We suggest that increased endogenous JA and possibly ABA are both evidence of, and assist in, acclimation of the *Arabidopsis* plants to everyday microstresses and improved plant growth. The elevated levels of fumaric acid, which is an intermediate of the tricarboxylic acid (TCA) cycle and a source of energy for cellular metabolism and plant growth^64–66^, suggest improved energy production and utilization under BC addition. In addition, fumaric acid behaves as a temporary carbon sink, and can serve as a source of energy for mitochondria function^65,67^. Hence, the presence of biochar in the potting medium mediated an enhancement in energy production and mobilization. The interaction between JA or ABA and fumaric acid is not yet understood.

The oxidative (ROS) damage induced by the applied heat stress was manifested in several ways: (i) an increase in MDA, (ii) modifications in expression levels of stress-related genes, and (iii) increase in the oxidized form of roGFP. In a number of instances, levels of these oxidative stress markers were different in + BC(+ HS) versus –BC(+ HS) plants. As an example, although HS resulted in upregulation of the expression of the *ZnF* gene, the extent of upregulation was lower in + BC(+ HS) *Arabidopsis* shoots than in –BC(+ HS) shoots. This may indicate there was less need for the *ZnF* protective response in the + BC plants. The *ZnF* protein has a key function as transcription factor in protein biosynthesis, and plays a crucial role in multiple abiotic stress responses including HS^68^. In contrast, the expression of the *GST* gene decreased in –BC plants when exposed to HS, while there was no HS-induced change in *GST* expression in + BC plants here was. GST enzymes are affected by different environmental factors including HS and help the plant to maintain cellular redox homeostasis^69^. Decreased expression of the *GST* gene in the –BC(+ HS) treatment can explain the increase in oxidative status of the plant as seen by increased oxidized roGFP and MDA. In this case, the higher expression of *GST* gene in + BC(+ HS) relative to –BC(+ HS) plants may be attributed to enhanced cellular detoxification mechanisms, for instance, through the conjugation of glutathione with ROS-generated electrophiles and toxic substances. In contrast, the expression of *DNAJ*/*Hsp40* gene was not affected by BC, although it did increase in response to HS. The increase in expression of *DNAJ*/*Hsp40* during HS is indicative of enhanced activity of thermoresponsive signaling mechanisms.

The decrease in lipid peroxidation and oxidative status and improvement in photosynthetic efficiency in the BC-treated plants following HS are physiological evidence for BC-elicited rapid responses to HS in the acclimated plant. These three parameters are interconnected. A heat-induced increase in lipid peroxidation^70^ and reduction in chlorophyll fluorescence and F_v_/F_m_ in *A. thaliana* is known^71^. Lipid peroxidation is a biochemical marker for ROS-mediated injury that occurs via a series of reactions involving polyunsaturated fatty acids of the cellular membranes^72^. Levels of lipid peroxidation can also be correlated with photosynthetic efficiency because of damage to the chloroplast structure and organizational composition of lipids of thylakoid membranes^71^. Studies show a link between plant acclimation to HS and initial higher level of saturated fatty acids^73,74^. Moreover, during HS, plants adapt by decreasing their levels of unsaturated lipids^75^. These trends are similar to the effects of the BC observed here (increased octadecanoic acid in the + BC(–HS) treatment and decrease in the tri-unsaturated lipids under + BC(+ HS) treatments).

Chloroplasts are a major source for ROS production during normal metabolic activities^76^. *Arabidopsis* Pla-roGFP1 was previously used to monitor plastid redox potential by *in-vitro* methods^77,78^. In the current study, the redox status of the plastid was measured in planta. Monitoring the oxidized and reduced fluorescence of Pla-roGFP on whole live plants confirmed that HS disturbed the plastid redox balance and increased the oxidized roGFP -related fluorescence. Hence, the HS significantly increased the roGFP oxidation/reduction ratio, likely via increased production of ROS molecules in the chloroplast. BC reduced this ratio to a normal plastid redox potential. Increased oxidative damage in leaf tissue leads to photoinhibition as expressed by a decrease in the F_v_/F_m_ ratio^79^. The suppression of chloroplast oxidation in plants grown in BC-amended soil suggests that the presence of BC suppresses oxidative stress in the intracellular environment and helps maintain redox state homeostasis and better activity of the photosystem II. A recent study examined the impact of biochar and beneficial microbe inoculum on proteomics and growth of *Arabidopsis thaliana* in a heavily As and Pb contaminated soil^80^. The gene ontologies terms commonly characterizing all cluster-related subnetworks were mainly related to energy and primary metabolism, and in part to “oxidative photosynthetic carbon pathway”. Although that study lacked a comparative non-stress condition and involved toxicity rather than HS, their outcome is in line with the results herein, which point to an energy-related ‘microstresses’ early-acclimation mechanism and a ROS-related acute HS acclimation mechanism.

The hormones auxin, ABA, SA, cytokinins and JA were modified by HS and BC. These modifications enhance our understanding of the mechanisms of BC protection from HS. IAA, among other roles, regulates shoot elongation and stress adaptation responses^81,82^. Levels of IAA and its biologically inactive amino acid conjugates, IAGlu, IAAsp, and OxIAA, point to the plant physiological state at given environmental condition^83^. HS resulted in increased IAA levels. Perhaps this increase is due to enhanced synthesis of IAA during HS^84^ or to its release from IAGlu, since IAGlu levels were reduced following HS. The decreased level of IAGlu in BC-treated plants, in general, may indicate a BC-mediated depression of the activity of GH3 family enzymes that are involved in IAA conjugation^85^, and may explain the higher active IAA in BC-treated plants. Viger, et al.^86^ reported that *Arabidopsis* growing in biochar-amended media exhibited enhanced signaling of IAA and brassinosteroid. IAA levels were highest in the + BC(+ HS) plants, and could have contributed to their success in partially overcoming the deleterious effects of the stress on growth. The increase in IAA corresponds to reports of BC-mediated increases in endogenous IAA in salt-stressed bean seedlings^87^. *Arabidopsis* transgenic lines (*iaaM-OX*) expressing higher endogenous IAA and IAA pre-treated WT exhibited enhanced drought stress resistance compared with a triple mutant (*yuc1yuc2yuc6*) expressing a lower level of IAA and non-treated WT^88^. Graber et al.^89^ hypothesized that biochar humic-like substances may have hormone-like effects on *Arabidopsis* that affect their response to phosphorus, somewhat similar to the way soil humic-like substances are suggested to influence phosphorus availability to crops. Under sterile conditions, the purified biochar-derived humic-like substances extract impacted *Arabidopsis* root development: primary roots were same in all the treatments, while both root hair length and density were lower in extract-amended growing media under both sufficient initial phosphorus and starvation initial phosphorus conditions compared with non-amended control. Since the differences could not be attributed to nutritional or biotic effects, they concluded that biochar-derived humic-like substances altered the *Arabidopsis* root response to environmental phosphorus by hormone-like signaling^89^.

Endogenous SA levels followed a pattern similar to that of endogenous IAA levels, namely, increasing after HS, and exhibiting a maximum level after HS in BC-treated plants. The SA response is a classic example of priming by BC for rapid acclimation to HS^40^. This response was different from that of JA, which was generally elevated by the presence of BC irrespective of the acute stress. ABA has a known role in different abiotic stresses^90^. Increased ABA accumulation due to HS suggests it actively participates in alleviation of oxidative stress and induction of thermotolerance. Biochar-induced plant growth promotion and other physiological activity could also be related with the increased level of ABA. The data do not reveal any clear involvement of cytokinins in heat stress or biochar responses.

## Conclusion

Taken altogether, our results are suggestive that BC in the growing medium elicited an early acclimation state in *Arabidopsis* plants, enhancing their ability to cope better with everyday microstresses throughout its growing period. Early acclimation involves a boosted TCA cycle and elevated JA and ABA hormone levels. BC also primed the plants to respond effectively against the acute oxidative HS response. The early acclimation mechanism against microstresses employs different mechanisms from the acclimation mechanism against the acute HS, which was ROS-related. These changes can explain "The Biochar Effect", a term originally introduced to describe BC-mediated improvements in plant health, flowering, and growth due to factors other than nutrition, water, or soil structure. The finding that BC-elicited early-acclimation in plants contributes to enhanced protection against heat stress may pave the way to implementing biochar as a tool to help protect plants from the heating effects of climate change.

## Materials and methods

### Biochar

The biochar in this study is the same as previously used in Kumar et al.^47^. It was produced from cattle manure feedstock in a laboratory pyrolysis system equipped with electric heaters and controller. The pyrolysis cells were under continuous nitrogen (N_2_) flow (1.003 atmospheric pressure) set nominally at 0.2 L min^–1^ for the entire process. The controller automatically increases the flow rate if the temperature rises above the set point, bringing it back down to the set point. Homogenized and air-dried cattle manure feedstock was placed in the pyrolysis cells for 3 h at 110 °C to remove residual water. Following dewatering, temperature was increased to 300 °C for 2 h, then to 430 °C for 30 min, and finally to 450 °C for 6 h. The heating elements were then turned off while N_2_ continued to flow for an additional 24 h to cool the sample. Biochar prepared from the same batch was used for the biochar characterization and all plant pot experiments. Detailed characterization methods are reproduced from Kumar, et al.^47^ (see Supplemental Information), and key physical and chemical characteristics are reproduced here in Table 2.

**Table 2:** Physical and chemical characteristics of cattle manure biochar used in the pot experiments (on a dry weight basis).

Parameters	Biochar properties
pH	10.0
Electrical conductivity (EC) (µS cm^–1^)	7910
Carbon (g kg^–1^)	227
Oxygen (g kg^–1^)	114
Hydrogen (g kg^–1^)	10.6
Nitrogen (g kg^–1^)	12.8
Mineral (ash) content (g kg^–1^)	628
Specific surface area (m^2^ kg^–1^)	2200
Total Pore volume (m^3^ kg^-1^)	1 × 10^‒5^
Calcium carbonate equivalents (g kg^–1^)	163
Cation-exchange capacity (mmol kg^–1^)	488

Reproduced with permission from Kumar, et al.^47^.

### Pot experiment and plant growth conditions

*Arabidopsis thaliana* (L.) Heynh. plants used in this study was of Col-0 ecotype background or wild type (WT). *Arabidopsis* WT plant expressing roGFP in the plastids (pla-roGFP2)^46,91^ seeds used in this study were obtained from Dr. Lewis Feldman’s laboratory in the University of California, Berkeley, California^31^. Plants were grown in 10 cm pots containing peat that was previously mixed with biochar at 10 g Kg^‒1^ by dry weight (+ BC treatment) or without biochar (‒BC treatment). Biochar application rate was chosen based on previous studies that demonstrated generally beneficial plant responses to various biochar types in multiple plant/biotic stress systems^92,93^. Pots with 5 seeds each were incubated in the dark at 4 °C for three days before being transferred to a controlled condition growth chamber (CMP6050, Conviron, Winipeg, Manitoba, Canada) as follows: 23/21 ºC, 12/12 h day/night, 180 μmol m^−2^ s^–1^ photosynthetically active radiation (PAR). Each treatment was made up of three replicate pots^94^ and each pot is considered a biological replicate. Pots were maintained in the chamber for 21 days in trays continuously flooded with 1 cm of tap water. Just before the heat treatment stage, seedlings were culled from the pots to retain the best-developed specimen in each pot. Heat stress (+ HS) was applied by transferring pots to a continuous airflow oven at 50 ºC for 30 min, and non-heat stress (‒HS) control plants were placed at 22 ºC in the dark for the same length of time (mock stress) following method described by Joshi, et al.^28^. Experiments were repeated multiple times to validate reproducibility of the experimental system. The seed and biochar batches remained the same for all the experiments to assure comparability. Seed batch viability was confirmed by determining germination percentage each time. The average germination percentage was 87.5 ± 3.43.

The collection of plant material complied with institutional, national, and international guidelines and legislation concerning *Arabidopsis* transgenic plants. In accordance with guidelines and regulations for biosafety in Israel, an isolated growth room was used to grow and cultivate the *Arabidopsis* plants, and residues and water were discarded according to protocol.

### Plant growth parameters and leaf nutrient content

In some of the experimental repeats, plants were harvested after 21 d to determine above ground biomass fresh weight (FW). In other repeats, pots were replaced in the growth chamber under the same growing conditions as before the HS for an additional 10 d following the HS and mock stress treatments. Development of inflorescence (flowering) was determined by measuring the inflorescence height 10 days following the stress/mock stress treatment. Sampled shoots were washed, dried completely at 60 ºC, and ground into fine powder. Subsamples (0.1 g) were digested by microwave^95^ with 10 ml HNO_3_ for analysis of total zinc (Zn), copper (Cu), iron (Fe), manganese (Mn), calcium (Ca) and magnesium (Mg) by atomic adsorption spectroscopy (AAS, AAnalyst 400, Perkin Elmer, U.S.A.), and sodium (Na) and potassium (K) by flame photometer (M410, Sherwood Scientific Ltd., UK).

### Shoot organic acids and total phenols

Shoot organic acids from fresh material (50 mg) were extracted in 1 mL of 2.5% H_2_SO_4_ (v/v) in methanol in a Teflon screw capped glass tube following Li-Beisson, et al.^96^. Gas chromatography-mass spectrometry (GC–MS, model 6890 N/5973 N, Agilent, Santa Clara, CA; Agilent DB-FFAP capillary column, 30 m, 0.25 mm id) in scan mode was performed to identify compounds. Results were analyzed by comparing peak area normalized to the area of hexadecanoic acid. Shoot total phenols including polyphenols in freeze-dried *Arabidopsis* leaves (20 mg) were determined using the Folin Ciocalteu method^48^ with gallic acid as the standard.

### Photosystem II (PSII) maximal efficiency

Photosystem II (PSII) maximal efficiency of the targeted leaf (7^th^) of 21 d old plants was measured at room temperature immediately after the heat/mock heat treatment. Leaves were first dark-adapted for 30 min using leaf clips. Fluorescence intensities in the dark-adapted leaves were recorded using a portable Handy Plant Efficiency Analyzer (PEA-2126) fluorimeter (Hansatech Instruments Ltd., Kings Lynn Norfolk, UK) after illuminating with a saturating light intensity of 3000 μmol m^−2^ s^−1^ following method described by Sengupta et al.^97^.

### Lipid peroxidation

Lipid peroxidation in the fresh shoot (0.5 g) was estimated as MDA content using the thiobarbituric acid (TBA) reaction following Heath and Packer^98^. MDA was calculated using an extinction coefficient of 155 mM^‒^^1 ^cm^‒1^.

### Measurements of roGFP fluorescence by fluorometer and IVIS

In-vivo evaluation of plastid-specific redox potential was done with the pla-roGFP2 plants^91^. After 21 d growth, plants were transferred to the dark for 1 h. Fluorescence from the whole plant was then determined by excitation at 420 nm for oxidized and 480 nm for reduced forms, with emission determined at 515‒575 nm (set by GFP filter), using an IVIS® Lumina II Instrument (Caliper Life Sciences, Massachusetts, U.S.A.). The ratio of fluorescence emitted during excitation at 420 nm, versus the fluorescence emitted during excitation at 480 nm following reduction of basal fluorescence (420/480) describes the ratio of oxidized roGFP2 to reduced roGFP2. A higher 420/480 ratio indicates a more oxidized state, and a lower 420/480 ratio indicates a more reduced state. The IVIS was equipped with a highly sensitive, liquid N_2_-cooled charge coupled device camera (IS1330N6337, Andor, iKon). Fluorescence acquisition time was 2 s for 420 nm and 1 s for 480 nm and binning-8. Data was analyzed using Living Image® software (version 4.3.1; Caliper Life Sciences, Massachusetts, U.S.A.). The in-vivo measurement of whole plant redox potential using the IVIS method is the first of its kind. It has many advantages for determining ROS and redox state compared with conventionally used methods like staining with fluorescent dye^46^: non-destructive, real-time, amenable to time-series analyses, and lacking possibly confounding mechanical stress artifact. For comparison, conventional in-vitro measurements of roGFP fluorescence were done^91^. Leaf discs (0.5 cm diameter) were cut from the seventh leaf and floated on 200 μl milliQ water in 96 well plates with their abaxial side up, and kept in dark for 1 h. Fluorescence was measured using a fluorescence plate reader (Synergy TM2, BioTek Instruments Inc., Winooski, VT, U.S.A.) from the upper side. Leaves were excited by using 400 ± 15 nm for oxidation and 485 ± 10 nm filters for reduction, and emission was recorded using 528 ± 20 nm emission filter.

### Total RNA extraction, c-DNA synthesis and quantitative real time-polymerase chain reaction (RT-PCR) analysis

To analyze specific gene expression, plants shoots were frozen immediately in liquid nitrogen following the HS/mock treatment. Approximately 60 mg of plant sample was used for total RNA extraction by RNAqueous Phenol-free total RNA Isolation kit (Applied Biosystem-AM1912M) following the manufacturer’s protocol. RNA samples were treated with DNase inactivation reagent using TURBO DNA-free™ Kit (Applied Biosystem-AM1907). The RNA yield was above 70 ng/µL and quality (260/280) was between 1.7 and 2.3 assessed by Nanodrop. cDNA synthesis was carried out by mixing 10 μL of diluted (500 ng) RNA with the 10 μL of reverse transcription master mix from High-Capacity cDNA RT kit with RNAse inhibitor (Applied Biosystem-4374966) following manufacturer instructions. cDNA was stored at − 20 ºC until used for RT-PCR analysis.

Five genes to be considered abiotic stress-induced genes in *Arabidopsis* were selected for RT-PCR analysis: *DNAJ heat shock protein* (*Hsp40*, At3g08970), *Zinc finger* (*ANI-like*) *family protein* (*ZnF*, At3g28210), *cytochrome P450-like protein* (*CP450*, At4g37370), *glycosyltransferase* (*GTF*, AT2G43820), and *glutathione S-transferase* (*GST*, At2g29470)^99–102^. The genes' specific primers were designed following the TaqMan standard protocol. UBIQUITIN-CONJUGATING ENZYME gene (*PEX4*) was used as an internal reference gene. RT-PCR analysis was carried out using the TaqMan Gene Expression Master Mix (Invitrogen) in 96-well plates with a QuantStudio 12 k Flex Real-Time PCR System following a TaqMan program: 50 ºC for 2 min (incubation), 95 ºC for 2 min (enzyme activation), 40 cycles of 95 ºC for 1 s (denaturation), and 60 ºC for 20 s (annealing/extension). Details of the TaqMan assay protocol are given in the supporting information. All RT-PCR results were normalized using the C_t_ value corresponding to the reference gene. The relative expression levels of the target gene were calculated following the 2^–ΔΔCT^ method^103^. Results are a mean of three independent biological replicas. All PCR work was done using the service of the Center for Genomic Technologies, The Alexander Silberman Institute of Life Sciences, The Hebrew University, Jerusalem, Israel.

### Hormone analysis

Plant phytohormones in *Arabidopsis* leaves were determined following the method in Kumar, et al.^47^. Analytes included: cytokinins [t-Z, t-ZR, isopentenyladenine (iP), iPR], auxins and derivatives [IAA, IAAsp, IAGlu, indole-3-butyric acid (IBA, OxIAA, indole-3-butyryl-l-glutamic acid (IBGlu], SA, JA, and ABA. Shoots were flash-frozen and stored at ‒80 ºC until extraction. Powdered frozen plant material (200 mg) was extracted in a mixture of isopropanol:methanol:glacial acetic acid and analysed by liquid chromatography-mass spectrometry (LC–MS). Detailed extraction method, chromatographic and MS parameters are given in the supporting Information. Analysis was performed using Volcani Center Metalolomic Unit at Agricultural Research Organization, Volcani Center, Israel.

### Statistical analysis

Data presented corresponds to mean value ± standard error (S.E.) (n = 3). Two-way analysis of variance (ANOVA, Tukey–Kramer HSD test) was performed using JMP (JMP Pro 14 software, SAS Institute, NC) with BC concentration and HS as the main factors. One-way and two-way ANOVA were also conducted where applicable, all at a significance level (*α*) of ≤ 0.05.

## Supplementary Information

Supplementary Files
