# Supplementary material for: Distinctive in-planta acclimation responses to basal growth and acute heat stress were induced in Arabidopsis by cattle manure biochar
Source: Sci Rep. 2021 May 10;11:9875. doi: 10.1038/s41598-021-88856-7 (PMC8110981; doi:10.1038/s41598-021-88856-7)
Supplement: Supplementary file 1 — Supplementary Files [file 41598_2021_88856_MOESM1_ESM.docx]

**Supporting Information**

**Distinctive *in-planta* acclimation responses to basal growth and acute heat stress were induced in *Arabidopsis* by cattle manure biochar**

Abhay Kumar^1^, Haya Friedman^2^, Ludmila Tsechansky^1^, Ellen R. Graber^1^*

^1^Department of Soil Chemistry, Plant Nutrition and Microbiology, Institute of Soil, Water and Environmental Sciences, Agricultural Research Organization, Volcani Center, Rishon LeZion 7505101, Israel

^2^Department of Postharvest Science, Agricultural Research Organization, Volcani Center, Rishon LeZion 7505101, Israel

**Corresponding Author:** Ellen R. Graber

**E-mail:** ergraber@agri.gov.il

**Fax:** +972 3-960-4017

**Tel:** +972 3-968-3307

**Material and methods**

*Biochar Characterization*

Mineral (ash) content (three replicates) was determined by mass loss of oven dry (105 ºC) biochar after heating to 450 ºC in air for 18 h, followed by cooling to ambient conditions. Biochar elemental analysis (C, hydrogen (H) and nitrogen (N)) was determined in triplicate using an EA-1112 Elemental Analyzer (Thermo Finnigan, Cambridge, Massachusetts, U.S.A.), with oxygen (O) being calculated by difference. Electrical conductivity (EC) and pH were measured of the supernatant of 1:20 m:v oven dry biochar:milliQ water after shaking for 2 h. Cation exchange capacity (CEC) was determined according to the cartridge method in Graber, et al.^1^ Calcium carbonate equivalents (CCE) was determined following Horneck, et al.^2^ with small modifications. The mineral content (given as “ash”) was determined by calculating the weight loss on ignition following American Society of Testing and Materials method (ASTM #D-1762-84). Specific surface area (SSA) and the total pore volume (TPV) was determined with N_2_ sorption after degassing the samples for 12 h at 105 ºC. SSA was determined from multiple points in the pressure region P/P_0_ of 0.05–0.25 using the Brunauer-Emmett-Teller (BET) equation^3^. Total pore volume (TPV) was determined following the Gurvich rule following Lowell, et al.^4^. Biochar particles underwent surface structure examination and elemental analysis using an FEI NanoSEM, field emission scanning electron microscope (FESEM) equipped with energy dispersive X-ray spectroscopy (EDS), and a scanning transmission electron microscopy (STEM; JEOL ARM200F, JEOL Japan) coupled with an electron energy loss spectrometer (EELS) and an EDS detector and surface functional groups analysis using X-ray photoelectron spectroscopy (XPS, ESCALAB-250Xi, VG Scientific UK, East Grinstead, UK) as described previously^5,6,7^.

*Hormone analysis*

LC-MS analyses were conducted using UPLC-Triple Quadrupole-MS (Waters Xevo TQ MS, [Massachusetts, U.S.A.](https://www.google.com/search?safe=active&rlz=1C1CHBD_enIN910IN910&sxsrf=ALeKk02U7PxADEL0CvcKROXqKB8dsvsmTA:1616520008214&q=Milford,+Massachusetts&stick=H4sIAAAAAAAAAONgVuLUz9U3MMy1yC54xGjCLfDyxz1hKe1Ja05eY1Tl4grOyC93zSvJLKkUEudig7J4pbi5ELp4FrGK-WbmpOUXpego-CYWFycmZ5QWp5aUFAMAxY3vB10AAAA)). Separation was performed on Waters Acquity UPLC BEH C18 1.7 µm 2.1 × 100 mm column with a VanGuard precolumn (BEH C18 1.7 µm 2.1 × 5 mm). Chromatographic and MS parameters were as follows: for abscisic acid (ABA), auxins (indole-3-acetic acid (IAA), indole-3-acetylaspartic acid (IAAsp), indole-3-acetyl-l-glutamic acid (IAGlu), indole-3-butyric acid (IBA), oxindole-3-acetic acid (OxIAA), indole-3-butyryl-l-glutamic acid (IBGlu)) and cytokinins (*trans*-Zeatin (t-Z), *trans*-Zeatin riboside (t-ZR), isopentenyladenine (iP), isopentenyladenosine (iPR) analysis the mobile phase consisted of water (phase A) and acetonitrile (phase B), both containing 0.1% formic acid in the gradient elution mode. The solvent gradient program followed are detailed in **Table S1** and S**2**:

**Table S1.** The solvent gradient program for ABA, auxins, and cytokinins.

| **Time (min)** | **Phase A %** | **Phase B %** |
| --- | --- | --- |
| Initial | 95 | 5 |
| 0.5 | 95 | 5 |
| 14 | 50 | 50 |
| 15 | 5 | 95 |
| 18 | 5 | 95 |
| 19 | 95 | 5 |
| 22 | 95 | 5 |

The flow rate was 0.3 ml min^‒1^, and the column temperature was kept at 35 ºC.  ABA, auxins and cytokinins analyses were performed using the ESI source in positive ion mode with the following settings: capillary voltage 3.1 KV, cone voltage 30 V, desolvation temperature 400 ºC, desolvation gas flow 565 l h^‒1^, source temperature 140 ºC.  Quantitation was performed using MRM acquisition by monitoring the:

247/173, 247/187 for ABA, 253/206, 253/234 for d6-ABA, RT – 8.55

220/136, 220/202 for t-Z, 225/137, 225/207 for d5 t-Z, RT – 2.35

352/136, 352/220 for t-ZR, 357/137, 357/225 for d5 t-ZR, RT – 3.45

336/136, 336/204 for iPR, 342/137, 342/210 for d6 iPR, RT – 6.05

204/69, 204/136 for iP, 210/75, 210/137 for d6 iP, RT – 4.78

176/103, 176/130 for IAA, 181/106, 181/134 for d5 IAA, RT – 7.36

291/130, 291/134 for IAAsp, 297/134, 297/136 for DN IAAsp, RT – 5.35

305/130, 305/148 for IAGlu, 311/134, 311/150 DN IAGlu, RT – 5.79

192/128, 192/146 for OxIAA, RT – 5.13

333/130, 333/186 for IBGlu, RT – 8.11

204/144, 204/186 for IBA, RT – 10.26

**Table S2.** The solvent gradient program for SA and JA analysis.

| **Time (min)** | **Phase A %** | **Phase B %** |
| --- | --- | --- |
| Initial | 95 | 5 |
| 0.1 | 95 | 5 |
| 5 | 40 | 60 |
| 6 | 5 | 95 |
| 9 | 5 | 95 |
| 10 | 95 | 5 |
| 13 | 95 | 5 |

ESI source was in negative ion mode with the following settings: capillary voltage 3.5 KV, cone voltage 30 V, desolvation temperature 530 °C, desolvation gas flow 650 L h^‒1^, source temperature 150 °C.  Quantitation was performed using MRM acquisition by monitoring the 137/65, 137/93 for SA, 141/97 for d4 SA, RT – 3.94, 209/59, 209/165 for JA, RT – 4.65. Acquisition of LC-MS data was performed under MassLynx V4.1 software (Waters).

# *RT-PCR TaqMan Assay Protocol*

Real time-PCR analysis was done with TaqMan Gene Expression Assays (AB-4448892) using TaqMan™ Fast Advanced Master Mix (AB-4444557). Each cDNA after preamplification was tested with six assays.

Each combination of cDNA with assay was tested in triplicates.

1. Master mixes were prepared for each cDNA sample:

| **Components** | **1X** | **20X** |
| --- | --- | --- |
| DDW | 2.6 µL | 52 µL |
| TaqMan Gene Expression Master Mix (2X) | 5 µL | 100 µL |
| cDNA | 0.4 µL | 8 µL |
| Total Volume | 8.0 µL | 160 µL |

1. Each assay was diluted 1:4 with nuclease free water. A 7.5 μL of assay were mixed with 22.5 µL of nuclease free water. Master Mix (8.0 µL) were pipetted into 96-well plate followed by TaqMan Gene Expression Assay (20X) (2.0 µL) and except to NTC.
2. NTC Master Mix (8.0 μL) were pipetted followed by TaqMan Gene Expression Assay (20X) (2.0 μL). NTC Master mix was prepared for NTC wells:

| **Components** | **1X** | **7X** |
| --- | --- | --- |
| DDW | 3.0 μL | 21 μL |
| TaqMan Gene Expression Master Mix (2X) | 5.0 μL | 35 μL |
| Total volume | 8.0 μL | 56 μL |

1. Plate was covered with adhesive cover.
2. Plate was briefly centrifuged to spin down the contents and to eliminate any air bubbles.
3. Plate was run on QuantStudio 12K Flex PCR system following below mentioned program:

Program: default Taqman-fast profile:

| **Parameters** |  | **Program** | | | | | | |  |
| --- | --- | --- | --- | --- | --- | --- | --- | --- | --- |
|  |  | **Incubation** |  | **Enzyme activation** |  | **Denature** |  | **Anneal/Extend (40 cycles)** | |
| Temperature °C |  | 50 |  | 95 |  | 95 |  | 60 | |
| Time |  | 2 min |  | 2 min |  | 1 sec |  | 20 sec | |

**Results**

**Table S3.** Two-way ANOVA analysis of heat stress (HS) and biochar concentration (BC) and their interactions (HS × BC). Multiple comparisons were done with the Tukey–Kramer HSD test. Asterisk (*) sign denotes a signiﬁcant difference at **p* < 0.05; ***p* < 0.001, ^ns^- non-significant.

| **Parameters** | **HS** | **BC** | **HS × BC** |
| --- | --- | --- | --- |
| Inflorescence height | **0.012*** | **<0.001**** | 0.718 |
| Fumaric acid | **0.026*** | 0.201 | 0.149 |
| Total phenol | 0.626 | 0.316 | 0.596 |
| F_v_/F_m_ | **<0.001**** | **0.016*** | **0.014*** |
| Malondialdehyde | **0.002*** | **0.005*** | **0.016*** |
| In-vivo roGFP2 | 0.226 | **0.035*** | 0.061 |
| In-vitro roGFP2 | **0.011*** | **0.008*** | 0.623 |
| Trans-Zeatin | 0.773 | 0.228 | 0.628 |
| Trans-Zeatin ribozide | **0.002*** | 0.532 | 0.447 |
| Isopentenyladenosine | **0.002*** | **0.004*** | 0.281 |
| Indole-3-acetic acid | **0.004*** | **0.025*** | 0.379 |
| Indole-3-acetylaspartic acid | 0.021 | 0.264 | 0.929 |
| Indole-3-acetyl glutamic acid | 0.186 | **0.032*** | 0.335 |
| Oxindole-3-acetic acid | 0.173 | 0.237 | 0.352 |
| Salicylic acid | **0.039*** | 0.208 | 0.322 |
| Jasmonic acid | 0.811 | 0.034 | 0.643 |
| Abscisic acid | 0.072 | 0.301 | 0.141 |

**Figure S1**

**Figure S1.** Effect of absence (‒BC) and presence (+BC) of biochar on the plant organic (fatty) acids of *Arabidopsis thaliana* exposed to heat stress (‒HS and +HS) at 21 days. Data presented here are showing the relative peak area to hexadeconeic acid. (**A**) 9-Hexadecenoate; (**B**) 7,10-Hexadecadienoate; (**C**) 7,10,13-Hexadecatrienoate; (**D**) Octadecanoic acid; (**E**) 9-Octadecenoic acid; (**F**) 9,12-Octadecadienoic acid; and (**G**) 9,12,15-Octadecatrienoic acid. Columns (means ± S.E.) labeled by different lowercase and uppercase letters are signiﬁcantly (*p* < 0.05) different within the ‒BC and +BC groups at ‒HS and +HS treatment conditions, respectively. Asterisk denotes the significant difference at *p* ≤ 0.05 according to Tukey Kramer HSD test between the –BC and +BC at same heat treatment (either –HS or +HS) conditions.

**Figure S2**

**Figure S2.** Effect of absence (‒BC) and presence (+BC) of biochar on the relative expression of analyzed genes of *Arabidopsis thaliana* exposed to heat stress (‒HS and +HS) at 21 days. (**A**) *Cytochrome P450-like protein*; and (**B**) *UDP-glycosyltransferase*. Columns represents the mean value (n=3) and range (calculated by evaluating using the 2^‒ΔΔCT^ ± the standard error)^8^.

**References**

1 Graber, E. R., Singh, B., Hanley, K. & Lehmann, J. in *Methods of biochar analysis* (eds B. Singh, M. Camps-Arbestain, & J. Lehmann) (CSIRO, 2016).

2 Horneck, D. A., Hart, J. M., Topper, K. & Koepsell, B. *Methods of soil analysis used in the soil testing laboratory at Oregon state university*. Vol. SM 89 21 (1989).

3 Brunauer, S., Emmett, P. H. & Teller, E. Adsorption of gases in multimolecular layers. *J. Am. Chem. Soc.* **60**, 309-319, doi:10.1021/ja01269a023 (1938).

4 Lowell, S., E. Shields, J., Thomas, M. & Thommes, M. *Characterization of porous solids and powders: Surface area, pore size and density*. Vol. 1 (Springer, Dordrecht, 2004).

5 Joseph, S. *et al.* The electrochemical properties of biochars and how they affect soil redox properties and processes. *Agronomy* **5**, 322 (2015).

6 Archanjo, B. S. *et al.* Nanoscale analyses of the surface structure and composition of biochars extracted from field trials or after co-composting using advanced analytical electron microscopy. *Geoderma* **294**, 70-79, doi:http://dx.doi.org/10.1016/j.geoderma.2017.01.037 (2017).

7 Kumar, A., Joseph, S., Tsechansky, L., Privat, K., Schreiter, I.J., Schüth, C. & Graber, E.R. Biochar aging in contaminated soil promotes Zn immobilization due to changes in biochar surface structural and chemical properties. *Sci. Total Environ*. **626**, 953‒961 (2018).

8 Schmittgen, T. D. & Livak, K. J. Analyzing real-time PCR data by the comparative CT method. *Nat. Protoc.* **3**, 1101, doi:10.1038/nprot.2008.73 (2008).
